# Computing and Evaluating Relationships Between Equal and Differential Factor Weighting for Fundamental Movement Skills and Physical Activity with Guided Active Play During Childhood

**DOI:** 10.3390/children12121615

**Published:** 2025-11-27

**Authors:** Glory Madu, Victoria Kwong, Dusan Calic, Taylor Cleworth, Angelo Belcastro

**Affiliations:** 1 School of Kinesiology and Health Science, Faculty of Health, York University, Toronto, ON M3J 1P3, Canada; glory95@my.yorku.ca (G.M.); vickykwong88@gmail.com (V.K.); calic.dusan.pt@gmail.com (D.C.); tclewort@yorku.ca (T.C.); 2 Muscle Health Research Centre, Faculty of Health, York University, Toronto, ON M3J 1P3, Canada

**Keywords:** locomotor and object control skills, measurement, factor loadings, guided active play

## Abstract

**Background/Objectives:** The Test of Gross Motor Development (TGMD-2) totals assume equal weighting of the 12 locomotor (LOC) and object control (OC) skills, yet validation studies indicate differential contributions. The study compared equal- and differential-weighted scores for LOC and OC skills, with three fitness and two physical activity (PA) outputs during guided active play (GAP). **Methods:** Children’s (n = 82; 7.6 ± 1.5 years) TGMD-2 LOC and OC differential factor weights were estimated with Exploratory Factor Analysis (EFA) and compared to equal weights with multiple linear regression (two, five, and eight predictors) and Chi-square analyses. Predictor variables included fitness, BMI, sex, age stages, and PA assessed by energy expenditure (PAEE) and intensity (MVPA) estimated using accelerometry during 1 h GAP. **Results:** EFA supported a two-factor structure (variance explained = 51.1%) with ≥0.500 loadings for 9/12 skills. Differential- and equal-weighted LOC and OC scores showed varied contributions from individual skills. Multiple linear regression analysis showed similar explained variances (R^2^) of 53% (PAEE), 40% (MVPA), 31% (OC), and 14% (LOC) for equal or differential scores with eight predictors. Although β coefficients varied, going from two, five, and eight predictors, the impact of equal and differential weights was comparable. Chi-square analysis indicated high OC associated with MVPA (X^2^ (4) = 9.42, *p* ≤ 0.05), LP, and STR with PAEE. **Conclusions:** TGMD-2 outputs with equal- and differential-weighted scores are adequate for clinical/educational use, which show similar relationships with PA and HRF variables. Differential-weighted TGMD-2 scores comprise different contributions of movement skills and may hold promise for intervention studies focused on varied or target tasks and movement abilities.

## 1. Introduction

Motor competency involves independent and interdependent functions, including motor coordination, motor ability, motor fitness, motor proficiency, gross motor competency, and Fundamental Movement Skills (FMS) [1]. Developing motor competence during childhood is important for accomplishing fine and gross motor tasks that impact goal-directed movements required of daily living. Movement/motor skills are categorized into locomotor skills (LOC) and object control (OC)/manipulative skills and described as the building blocks of more complex movements [2]. To better understand the relationships between developing movement skill competency and complex movements, a model focused on developmental trajectories leading to health- and performance-related outcomes has been proposed [3]. The model hypothesizes synergistic and reciprocal bidirectional relationships among movement skill levels, perceived physical competence, components of health-related fitness (HRF) and physical activity (PA) participation, which are stage- and sex-related during childhood [3]. It is hypothesized that during early childhood (EC), PA participation, improvements in HRF, and perceived physical competence contribute to gains in FMS levels. During middle-to-late childhood (MC-LC), it is hypothesized that proficiency gains in FMS contribute to complex movement patterns and PA participation that support positive health-related and performance/sport-related outcomes [1,3,4].

Of the several instruments assessing movement skills, the Test of Gross Motor Development-2 (TGMD-2), Movement Assessment Battery for Children-2 (MABC-2), and Bruininks-Oseretsky Test of Motor Proficiency (BOT-2) have been widely used in research, education, and clinical settings [1,5,6]. Whether the revised TGMD-3, with its focus on a general motor skills factor and specific factors for LOC and ball skills, supersedes the use of the TGMD-2 in the future is to be determined [7,8]. Notwithstanding the future potential of TGMD-3, the challenges and inconsistencies identified for interpreting LOC and OC scores when comparing relationships with behavioral and biological variables may persist [9]. Specifically, TGMD-2 validation studies show that the 12 movement skills have differential factor (skill) weights; however, the practice of computing summed LOC and OC scores assumes equal factor (skill) weights [5,10,11,12]. This methodological approach to applying skill factor weights may bias relationships between dependent and independent variables [13]. Although several reports have identified the relationships between equal-weighted total, LOC, and OC scores with PA and components of HRF [1,2,3,4], investigations of differential-weighted LOC and OC scores and PA and HRF are lacking in the literature [11,14]. Previous reports showing considerable variability for differential weights of TGMD-2 skills may be responsible for the gap in the literature [11]. For example, the range of differential factor weights reported for running includes 0.20, 0.45, 0.71, and 0.94 [10,12]. Differences in factor weights have been attributed to inappropriate or mis-specified approaches in estimating factor weights, with Exploratory Factor Analysis recommended over Confirmatory Factor Analysis for TGMD-2 outputs [11,13]. Specifically, Exploratory Factor Analysis using Maximum Likelihood factor extraction with oblique rotations provides an opportunity to explore how each skill loads on both factors. Furthermore, it is important to determine if the factor weights for LOC and OC skills align with the two-factor hypothesis for the TGMD-2 [5] prior to considering further statistical analysis [13,15]. While equal- and differential-weighted scores assess the same movement skills (LOC and OC), it is uncertain if the differential-weighted scores show similar relationships to equal-weighted scores.

To investigate the multiple linear regression relationships for LOC and OC skills with HRF and PA outputs, it is important to assess individual and/or grouped predictors since relationships between a predictor and the outcome may vary with increased predictors. Specifically, adding predictors can change relationships in many ways, including masking and/or mediating independent variable effects, re-evaluating the individual contribution of a predictor, and adjusting for multicollinearity and/or confounding variables [16,17]. Studying relationships between motor skills, fitness, and physical activity demonstrates how adding new variables reveals a more complex picture [18]. Therefore, the analysis of equal- and differential-weighted LOC and OC skills should include comparisons of many individual and/or groups of predictors (i.e., fitness, sex).

The concern that equal-weighted LOC and OC scores may mask differential factor weights has also been approached by categorizing high scores with relevant cut-off thresholds [13]. An investigation using cut-off thresholds to classify high LOC and OC levels in youth found significant associations between high movement skill categories and HRF [19]. As well, results for children (7–12 years) showed that cut-off categories for high movement skills not only align with motor skill proficiency but a significant association was observed with high levels of PA participation [20]. Furthermore, using cut-off thresholds for TGMD-2 skills has proven invaluable for categorizing childhood FMS levels from 25 countries across six continents [21]. However, cut-off thresholds applied to equal-weighted LOC and OC sum scores may not represent the characteristics of the measurement instrument, which might misclassify “high-level” classifications [13]. Individual averaged scores rather than summed scores may preserve the test metrics [13]. Investigation of high categories for LOC and OC scores and/or individual averaged scores for LOC (LIA) and OC (OIA) scores with PA outputs and HRF have yet to be reported. In addition, whether associations between high LOC and OC classifications and PA and HRF share a similar pattern of results compared to differentially weighted LOC and OC skills with PA and HRF is uncertain.

Previous studies using habitual and/or school-based PA with their sporadic bouts of different PA domains may provide too few opportunities for LOC and OC skills, thereby impacting the relationships with PA outputs [1,22,23]. It has been suggested that the relationships between LOC and OC scores and PA outputs may be impacted by the choice of locations used to capture children’s PA participation. In contrast, guided active play (GAP) has been suggested to promote opportunities for more varied activities for movements requiring combinations of LOC and OC skills [24,25,26]. While equal- and differential-weighted scores assess the same movement skills (LOC and OC), it is uncertain if the different weights show similar relationships with complex or challenging physical activities. Furthermore, whether guided active play (GAP)-based PA outputs for cooperative social games show similar relationships between PA with equal- and differential-weighted LOC and OC scores remains uncertain [27,28].

The purposes of this study were to (1) investigate if equal- and differential-weighted LOC and OC scores modify the nature of the relationships with guided active play PA and HRF; and (2) examine associations among high LOC, high OC skills, high guided active play PA outputs, and high components of HRF across childhood. Our objectives were the following: (a) compute differential-weighted LOC and OC scores by obtaining differentiated factor weights for LOC and OC using two-factor Exploratory Factor Analysis; (b) conduct multiple linear regression analysis of two (FMS and PA), five (+HRF) and eight (+sex, BMI, and age stages) predictor models to compare the influences of equal- and differential-weighted LOC and OC scores. Specifically, predictors included Physical Activity Energy Expenditure (PAEE), moderate to vigorous PA (MVPA), Aerobic Power (AP), Leg Power (LP), average grip strength (STR), sex and body mass index (BMI); (c) evaluate the associations between relevant cut-off thresholds for high categories of LOC, LIA, OC, and OIA skills with high PAEE, MVPA, and HRF variables; and (d) identify if equal- and differential-weighted LOC and OC scores and categorized LOC, LIA, OC, and OIA skills demonstrate shared relationships with PA and HRF.

It is hypothesized that (a) differential-weighted LOC and OC scores would demonstrate different contributions of movement skills to LOC and OC scores; (b) differential-weighted LOC and OC scores would demonstrate different relationships with PA and HRF outcomes compared to equal-weighted scores using two, five and eight predictors; and (c) high LOC and OC levels would be significantly associated with higher PAEE, MVPA, and HRF components across childhood.

## 2. Materials and Methods

### 2.1. Participants and Study Design

Children ranging from 5 to 10 years old registered in a seven-week community-based summer day camp program (subsidized fees) were recruited to participate in the study. Following an orientation session, 70% of parents/guardians provided their children’s PA readiness survey and informed consent for their children. Children excluded from the study were those not completing all the assessments (12%) and those with incomplete consent information (18%). All procedures in this study were supported by the community center and approved by the University’s Human Participation Research Ethics Committee prior to the start of the study.

The study was conducted within a community-based summer day camp program scheduled for 7 weeks (4 d wk^−1^) from 8:30 am–3:30 pm (exclusive of weekends). Children completed two trials of anthropometric, health-related fitness, and FMS measurements separated by two days in an air-conditioned gymnasium. Children’s PA data was collected during a 55 min guided active play (GAP) program using cooperative (social) games, such as Fishes and Whales, What time is it Mr. Wolf, Clothespin tag, Crocodile-Crocodile, and Arches tag, framed by a warm-up (i.e., static/dynamic stretches) and cool-down [26,27].

### 2.2. Anthropometric and Health-Related Fitness

Body mass and standing height were determined using an electronic scale and a stadiometer, with body mass index (BMI) calculated as previously described [26,29]. Regarding health-related fitness parameters, Aerobic Power (AP), expressed as VO_2_max, was estimated from a multistage 20 m shuttle run using the speed of the last stage completed and age (VO_2_max = 31.025 + 3.238 (Speed) − 3.248(Age) + 0.1536.Age × Speed) [30]. The 20 m shuttle run test has demonstrated moderate to high construct validity (r = 0.78) for measuring VO_2_max when compared to direct gas analysis and high test–retest reliability (r = 0.89) [30,31,32]. Leg Power (LP), expressed in Watts, was derived from vertical jump height (VJH), calculated as the difference between standing reach and jump reach (cm), and body mass (kg) (W = 54.2 × VJH + 34.4 × body mass − 1520.4) [33]. Strength (STR) was measured using a hand grip dynamometer and expressed in kilograms (kg). Handgrip strength was measured in two trials per hand, performed alternately. The mean score for each hand was then combined to obtain the overall grip strength STR score [27]. All HRF variables were assessed by experienced university students who had demonstrated a coefficient of variability (+/−3%) for each method.

### 2.3. Fundamental Movement Skills

The Test of Gross Motor Development 2 (TGMD-2) was used to assess gross motor quotient (GMQ), locomotor skills (run, hop, leap, horizontal jump, slide, gallop), and object control skills (striking, kicking, dribbling, catching, throwing, rolling) as outlined in TGMD-2 Examiner’s Manual [5]. Performance criteria for each skill were assessed during two test trials. If a child demonstrated the performance criterion, they received a score of 1; if not, a 0 was recorded. FMS mastery was achieved if all performance criteria were met, and FMS proficiency was obtained if all but one of the performance criteria were met [5]. Kinesiology students trained in administering the TGMD-2 tests completed all assessments in small groups (≤4) of children. Prior to the study, intra-rater reliability for each rater (kinesiology student) was assessed on four or more randomly assigned videotaped trials of six children performing the TGMD-2 test over 8 days. Inter-rater reliability was assessed between 10 raters measuring the same 10 children performing at least 10 or more TGMD-2 tests over 8 days. The intra-rater and inter-rater reliabilities were strong, with ICC = 0.97 (CI 0.94 to 0.99) and ICC = 0.93 (CI 0.90 to 0.96), respectively. Furthermore, the variability in FMS scoring for all assessors against standardized tasks was determined by comparing scores with those from an expert (i.e., PhD with 10 years of experience with children’s assessments).

### 2.4. Physical Activity

Children’s PA was quantified and characterized with accelerometers (ACC; ActiGraph GT3X+, Pensacola, FL, USA) worn on the right hip during each GAP session (55 min). The sampling interval was set at a 10 s epoch with a frequency of 30 Hz, and ActiLife v6.1 software was used to calculate ACC vectors expressed in arbitrary units (counts per 10 s). Physiologically relevant outputs were determined using vector outputs to estimate oxygen consumption (VO_2_) (mLO_2_ kg^−1^ min^−1^) with a regression equation (y = 0.0025 (V) + 2.2266) derived from children’s games [26], which were used to calculate energy expenditure (EE) during the GAP sessions. For estimated VO_2_, the explained variance (R^2^) and Standard Error of Estimate (SEE) using specific laboratory-based equations were 0.95 and 1.07 mLO_2_ kg^−1^ min^−1^, respectively. Furthermore, physiologically estimated metabolic equivalents (MET) were determined using a regression equation (y = 0.0045 (V) + 0.9912) generated for GAP games. The METs were used to classify PA intensity as sedentary (≤1.9 MET), very light (2.0–2.9 MET), light (3.0–3.9 MET), moderate (4.0–5.9 MET), and vigorous (≥6 MET) [19,21]. Intensity data reported are expressed as a percentage of PA time spent at moderate and vigorous PA (%MVPA; ≥4 MET).

### 2.5. Statistical Analysis

Descriptive statistics (mean, standard deviation, skewness, and homogeneity of variances) were determined for anthropometric, FMS, HRF, and PA variables using SPSS v29. TGMD-2 individual movement skills were summed into Total (LOC + OC), LOC, and OC subtypes, assuming equal (E) weighting of each skill. The percentage of contribution of each LOC and OC skill to the summed score was determined. Differential factor (DF) weights were obtained with Exploratory Factor Analysis (EFA) and a two-factor solution using Maximum Likelihood extraction and oblimin rotation with Kaiser Normalization. These factor weights were used to compute differential-weighted Total, LOC, and OC scores. EFA of TGMD-2 data showed that factor loadings had a strong fit for further factor analysis as evidenced by the Kaiser–Meyer–Olkin measure (0.83) of sample adequacy and a significant Bartlett’s Test of Sphericity (*X*^2^ (66) =186.83, *p* < 0.001) and a goodness-of-fit test (*X*^2^ (43) =35.706, *p* = 0.777). Our cohort comprised girls and boys ranging from 5 to 7 years (early childhood) and 8-to-10 years (primary school children), since they influence motor skill scores and may have limited the stability and generalizability of our results. As such, two approaches were used to improve the stability and generalizability of the results. These included (a) comparison of estimated EFA factor loadings using a split sample; and (b) adjusting for sex, BMI, and stage covariates with multiple linear regression analysis of E and DF weighting scores on the dependent variable. A split-sample cohort was prepared using stratified sampling of two stages for children in the current sample (n = 40) and for children from previous studies (n = 42) [27]. Split-group analysis showed comparable statistics for the Kaiser–Meyer–Olkin measure (0.822), Bartlett’s Test of Sphericity (*X*^2^ (66) = 294.184, *p* < 0.001), and goodness-of-fit test (*X*^2^ (43) = 46.004, *p* = 0.349). Power analysis showed that a sample size of 86 was necessary to achieve a power level of 0.80 with an alpha level of 0.05, an effect size of 0.42 (based on factor loadings for 12 observed variables and 12 latent variables) [34,35]. Differential-weighted LOC and OC skills were obtained by multiplying individual motor skills by the weighted estimates. The differential-weighted scores for LOC and OC skills, and the percent contribution of each differential weighted skill, were determined for all children, girls and boys. The LOC and OC equal- and differential-weighted scores and the 12 individual scores were compared using Analysis of Variance (ANOVA), as well as paired and independent *t*-tests with SPSS v29. Power analysis for *t*-tests (n = 82) showed a power level of 0.8933 with an effect size of 0.35 and an alpha level of 0.05 [36]. For ANOVA, the within and between interactions showed a power level of 0.999 with an effect size of 0.33 and an alpha level of 0.05 [36].

Multiple linear regression was used to examine the reciprocal relationships between LOC and OC equal- and differential-weighted scores and PAEE and MVPA. Regression models examined reciprocal relationships between LOC and OC equal- and differential-weighted scores with PAEE and MVPA (two predictors). Second, the impact of AP, LP, and STR variables was included (a total of five predictors), followed by the addition of sex, BMI, and stage (a total of eight predictors). Sex was represented with girls as the reference group, and stage was represented with early childhood as the reference group. Also, model fit was assessed with the F-statistic (F), as well as the total variance accounted for (R^2^) and the adjusted variance accounted for (aR^2^). Standardized beta (β) coefficients for all variables were assessed at alpha levels, *p* ≤ 0.05, *p* ≤ 0.01, and *p* ≤ 0.001. A post hoc power analysis was conducted using G*Power 3.1 [36] to assess the adequacy of the sample size for the multiple linear regression (n = 82) indicated a power level of 0.99 with eight predictors, an effect size of (0.68), and an alpha level of 0.05.

Categorization of TGMD-2 LOC and OC skills, PA, and HRF variables was classified by cut-off thresholds between low (≤33%), medium (33–66%), and high (≥66%) categories. ANOVA and Tukey’s post hoc test assessed the mean differences between tertiles for LOC, OC, PAEE, MVPA, and HRF variables at *p* ≤ 0.05. The associations were assessed with a 3 × 3 Chi-square Fisher Exact test and Likelihood Ratio for small sample sizes (<50) and small cell sizes (<5). Cramer’s V statistic was used to assess the strength of a relationship between two categorical variables, as small (≤0.15), medium (>0.15 and <0.25), and large (≥0.25) [37]. Statistical comparisons of cross-tabulation analysis involved calculating z-scores from standardized residuals to determine alpha levels when comparing high and low classifications. The significance (*p* ≤ 0.05) levels were adjusted with the Bonferroni method to account for the number of statistical comparisons performed. Power analysis for Chi-square tests (n = 82) showed a power level of 0.721 with degrees of freedom (4), moderate effect size (0.325), alpha level of 0.05, and a Critical *X*^2^ = 9.043 [36]. All analyses were conducted using SPSS v29, with statistical significance of *p* ≤ 0.05, unless stated otherwise.

## 3. Results

### 3.1. Descriptive and Pearson Correlation Results

Children’s anthropometric, fitness, and FMS characteristics (mean and SD) are presented in Table 1. Children with a mean age of 7.6 ± 1.5 years were partitioned into age stages I (5–7 years) and II (8–10 years). Significant differences across the age stages were observed for Aerobic Power (AP) (F (1, 78) = 17.4, *p* ≤ 0.001), with children in stage I showing higher AP scores than stage II children. There was a significant difference across the age stages and between girls and boys for Aerobic Power (AP). AP decreased with age stages overall and more for girls, while boys were relatively stable (F (1, 78) = 9.2, *p* ≤ 0.001). Physical Activity Energy Expenditure (PAEE) (F (1, 78) = 17.2, *p* ≤ 0.001), Leg Power (LP) (F (1, 78) = 11.3, *p* ≤ 0.001), and grip strength (STR) (F (1, 78) = 296.9, *p* ≤ 0.001) significantly increased with age stages. Sex differences for moderate-vigorous physical activity (MVPA) showed that boys had significantly more intensity than girls across all age stages (F (1) = 18.8, *p* ≤ 0.001). Boys demonstrated higher object control (OC) skills than girls (F (1, 78) = 13.4, *p* ≤ 0.001) during age stages I and II (F (1, 78) = 5.1, *p* ≤ 0.05). Differences across age stages and between girls and boys for BMI and LOC scores were not statistically significant (*p* > 0.05).

Pearson correlations showed that LOC and OC were not significantly related to PAEE (r = 0.15 and r = 0.19, *p* > 0.05). In contrast, MVPA positively correlated with LOC (weak effect, r = 0.28, *p* ≤ 0.05) and with OC (moderate effect, r = 0.36, *p* ≤ 0.01). MVPA was strongly related to PAEE (r = 0.67, *p* ≤ 0.01). Based on the correlation analyses, PAEE was moderately related to LP (r = 0.55, *p* ≤ 0.01) and STR (r = 0.51, *p* ≤ 0.01). MVPA was moderately related to AP (r = 0.45, *p* ≤ 0.01) (Table 2).

### 3.2. Exploratory Factor Analysis of the Test of Gross Motor Development-2

Exploratory Factor Analysis identified two factors that explained 51.1% of the variance. The rotated factor loadings showed a strong pattern grouping of OC skills with Factor 1 and LOC skills with Factor 2 (Table 3). Although two skills, run and hop, showed weaker loadings on Factor 2, they were included with Factor 2 to coincide with the two-factor structure model [5]. The highest factor weight estimates for each movement skill were used to compute differentiated Total, LOC, and OC skill scores. Positive relationships were observed between equal- and differential-weighted scores for Total (r = 0.998, *p* ≤ 0.001), LOC (r = 0.995, *p* ≤ 0.001), and OC (r = 0.996, *p* ≤ 0.001) scores. A comparison of the means for equal- and differential-weighted LOC and OC scores showed significantly lower LOC and OC skills scores for the differentiated-weighted skills (Table 4). Boys were found to have higher total and OC scores than girls (*p* ≤ 0.05). Girls and boys showed non-significant differences for equal- and differentiated-weighted LOC scores.

### 3.3. Comparison of Equal Weights and Differential Weights for Individual LOC and OC Skills

**Figure 1:**
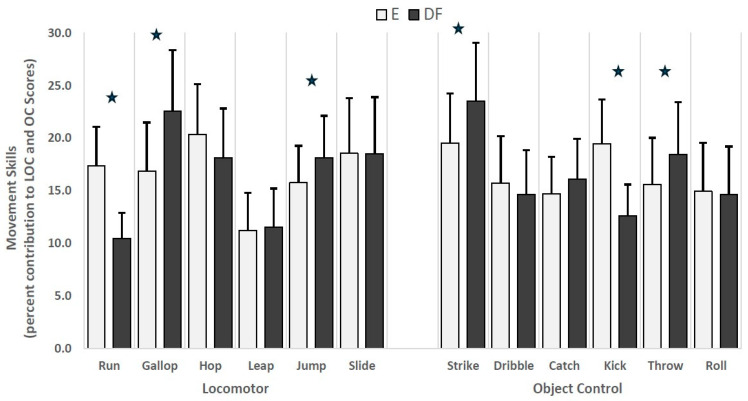
Percent contributions of locomotor and object control movement skills’ equal and differential weightings. The “star” denotes statistical significance for equal- and differential-weighted skills at *p* ≤ 0.05. The differential factor weighting for each movement skill was determined by multiplying the LOC and OC raw scores by their assigned weight following Exploratory Factor Analysis using Maximum Likelihood extraction and oblimin rotation with Kaizer Normalization.

The impact of factor weights assessed by percent contribution of individual equal and differential weighted LOC and OC skills relative to the LOC and OC subtype scores is presented in Figure 1. Overall, the percentage of equal-weighted skills ranged from 11.5 ± 4.4% (leap) to 20.4 ± 5.3% (hop) and DF skills from 10.3 ± 2.3% (run) to 22.6 ± 6.3% (gallop) for LOC scores. Considering differential-weighted skills, the gallop (t = 42.46, *p* ≤ 0.05) and jump (t = 33.98, *p* ≤ 0.05) showed significantly higher contributions to the LOC score, while the contribution of the run skill was lower by 7% (t = 48.43, *p* ≤ 0.05). Analysis of girls and boys showed similar differences for the percent contributions when comparing equal and differential-weighted LOC skills.

The percent contributions for OC skills showed equal-weighted skills ranging from 14.5 ± 3.0% (catch) to 19.5 ± 5.5% (strike) and differential-weighted skills from 12.3 ± 2.4% (kick) to 23.6 ± 5.9% (strike). Differential-weighted strike (t = 39.03, *p* ≤ 0.05) and throw (t = 39.69, *p* ≤ 0.05) skills showed significantly higher contributions to the OC score, while kick showed a lower contribution of 6.5% (t = 48.99, *p* ≤ 0.05) to the OC score (Figure 1).

### 3.4. Multiple Linear Regression Analysis for Equal- and Differential-Weighted LOC and OC Skills with Physical Activity and Health-Related Fitness

Regression analysis of equal- and differential-weighted LOC and OC scores with PAEE and MVPA was compared with the F-statistic, R^2^, and aR^2^ (Table 5). For all models, the use of differential-weighted skills for LOC and OC scores displayed comparable model fit statistics observed for equal-weighted skills. Generally, increasing the number of predictor variables from two (LOC, OC) to eight (LOC, OC, AP, LP, STR, sex, BMI, and stage) showed larger F-statistics and larger aR^2^ from 6% to 41% for PAEE (*p* ≤ 0.001) and from 12% to 34% for MVPA (*p* ≤ 0.001) (Table 5). When OC was the dependent variable, a similar pattern was observed for two (PAEE, MVPA) and eight (PAEE, MVPA, AP, LP, STR, sex, BMI, and stage) predictors, with the aR^2^ going from 13% to 27%. Predicting LOC with 2 to 8 predictors showed a lower trend, with F-statistics reporting 8% for 2 predictors to an aR^2^ of 3% for 8 predictors. Overall, results showed a positive reciprocal relationship from MVPA to OC and from OC to MVPA with R^2^ values of 31% and 40% for each pathway. Conversely, a significant positive relationship was evident for PAEE to LOC (R^2^ of 53%, *p* ≤ 0.001), while the reverse pathway was not significant (R^2^ of 14%, *p* > 0.05). The relationships identified for equal- and differential-weighted LOC and OC scores as either dependent or independent variables were comparable when considering two-, five- and eight-predictor models (Table 5).

Standardized beta (β) coefficients were compared for equal- and differential-weighted skills to better understand the explanatory power of regression models with varying numbers of predictors. In two-predictor models for PAEE and MVPA using equal-weighted LOC and OC skills, OC showed significant relationships with PAEE (β = 0.339, *p* ≤ 0.05) and MVPA (β = 0.283, *p* ≤ 0.05), while LOC skills were not significant. When fitness predictors (i.e., AP, LP, and STR) were included, the relationships between motor skills and PA outputs were no longer significant. However, children’s LP (β = 0.386, *p* ≤ 0.001) was related to PAEE, and AP (β = 0.441, *p* ≤ 0.001) influenced MVPA. When the 5-predictor model included adjustments for sex, BMI, and stage (early childhood and primary school children), the motor skill predictors did not significantly predict PA outputs. Adjusting for stages revealed non-significant relationships to PA outputs; in contrast, BMI was significantly related to PAEE (β = 0.316, *p* ≤ 0.001), while sex showed boys having a relationship to MVPA (β = 0.351, *p* ≤ 0.001) compared to girls. The relationships identified for equal- and differential-weighted LOC and OC scores predicting PAEE and MVPA outputs were consistent across the two-, five-, and eight-predictor models (Supplementary Tables S1 and S2).

Multiple linear regression analysis examining LOC and OC scores as dependent variables, with PA outputs, fitness, sex, BMI, and stages as predictors, revealed that among the five predictors (PAEE, MVPA, AP, LP, and STR), only MVPA (β = 0.304, *p* ≤ 0.05) had a statistically significant effect on LOC. The other predictors were not significant (*p* > 0.05). MVPA did not predict OC scores; however, when adjusting for sex, results showed that boys were significantly related to OC scores (β = 0.345, *p* ≤ 0.05), but not developmental stage and/or BMI. (Supplementary Table S3). Furthermore, the prediction of movement skills scores was consistent across the two-, five-, and eight-predictor models (Supplementary Tables S3 and S4).

### 3.5. Associations Between High and Low Classifications for PA Outputs, Components of HRF, Summed LOC and OC Skills, and Individual Averaged LOC (LIA) and OC (OIA)

A summary of the Chi-square Fisher Exact analysis for FMS, PA, and HRF variables is presented in Table 6. Significant associations were observed between MVPA with OC (X^2^ (4) =9.42; *p* ≤ 0.05) and OIA (X^2^ (4) =9.55; *p* ≤ 0.05), where 37% of the high MVPA group were overrepresented by children from the high OC and high OIA categories. Of the 12 motor skills, only the high dribble showed a significant association with high MVPA (X^2^ (4) = 11.72; *p* ≤ 0.05), with 41% of the children overrepresented in the high MVPA (Supplementary Material). Non-significant associations were found between MVPA with LOC and LIA skills. Non-significant associations were found between PAEE and LOC, OC, LIA, and OIA skills.

Examination of PA outputs and HRF showed significant associations between PAEE with LP (X^2^ (4) =14.04; *p* ≤ 0.05) and STR (X^2^ (4) =15.51; *p* ≤ 0.05) (Table 6). Specifically, the high PAEE group (>221 kcal 55 min^−1^) was overrepresented by children in the high LP (52%) and high STR (56%) groups. Significantly more boys with high PAEE were associated with the high LP and high STR groups. Conversely, the low PAEE group was overrepresented by 57% of the low LP group. Furthermore, a significant association was observed between MVPA and AP (X^2^ (4) =14.55; *p* ≤ 0.05), with low MVPA overrepresented by 57% in the low AP category (Table 6).

Investigation of LOC and OC skills with components of HRF showed significant associations between OIA and STR (X^2^ (4) =11.21; *p* ≤ 0.05). Cross-tabulation analysis showed that children in the high OIA category were overrepresented by 42% more children with high STR (>24.1 kg), while LOC associations were non-significant. Boys showed a significant association between OIA and STR (X^2^ (4) =13.00; *p* ≤ 0.05) compared to girls in the high OIA group, with an overrepresentation (45%) from the high STR group. LOC, OC, LIA, and OIA showed non-significant associations with AP and LP. Of the 12 motor skills, only the high throw showed a significant association with high LP, with 50% of the children overrepresented in the high LP group (X^2^ (4) =11.40; *p* ≤ 0.05) (Supplementary Material).

## 4. Discussion

The purpose of this study was to examine the relationships between TGMD-2 outputs computed with equal and differential weights with PA outputs, HRF, sex, and BMI. LOC and OC scores using differential motor skill weights obtained from a two-factor Exploratory Factor Analysis were found to have a strong linear relationship with equal-weight motor skill scores. Multiple linear regression revealed good model fit statistics for equal- and differential-weight LOC and OC scores. Equal- and differential-weighted LOC and OC scores showed similar relationships when predicted with PAEE, MVPA, AP, LP, STR, sex, BMI, and stage. β coefficients for equal- and differential-weighted LOC and OC skills each confirmed significant bidirectional effects between OC skills and MVPA across childhood. In addition, novel one-way relationships between OC on PAEE and MVPA on LOC were observed with guided active playing of cooperative games. Comparison of β coefficients across two, five, and eight predictors showed suppressed effects by AP, LP, sex, BMI, and stage on PA outputs that were consistent for both equal- and differential-weighted LOC and OC skills. To the best of our knowledge, this is the first study to calculate and compare different percentage contributions of movement skills for LOC and OC scores using equal- and differential-weighted factors. Analysis of high LOC and OC categories showed findings comparable to results with equal- and differential-weighted skills. In summary, positive relationships between LOC and OC skills with PA outputs and HRF were consistent across three weighted approaches for computing TGMD-2 scores. In conclusion, equal-weighted scores provide valuable clinical and educational information about children’s gross motor competence. However, differential weighting shows promise of a more comprehensive description of the relationship between individual movement skills and the types of movement activities with PA outcomes.

Previous reports supporting a two-factor structure model for TGMD-2 LOC and OC scores are theoretically justified [5,10,11]. While summing LOC and OC skills provides a broad assessment of overall gross motor competency, this approach does not consider differential factor weightings with estimated cross-loadings and latent variances for LOC and OC skills [11]. Although Exploratory Structural Equation Modeling is preferred, our study adopted a two-factor Exploratory Factor Analysis that explained 51% of the variance, showing moderate-to-strong standardized beta (β) coefficients (>0.500) for 9 out of 12 movement skills. Although two skills, run and hop, revealed lower beta (β) coefficient estimates for Factor 2 than with Factor 1, based on the two-factor structure validated for the TGMD-2, Factor 2 estimates were used to determine differential weighting. The process of assigning individual skills with lower estimates to align with the TGMD-2 subtypes has been used in previous studies [10,11,12]. Because factor loadings are sensitive to extraction and/or rotation methods, individual factor loadings may not be an accurate representation of the differences among factors [11,13]. Also, computing scores with differential weights may not conserve characteristics of factors identified in the original data [13,15]. It was reported that a linear relationship of r = 1.0 between equal- and differential-weighted data found is indicative of a preserved structure from the original assessment [15]. Accordingly, the near-perfect strong positive linear relationship of r = ≥0.98 between equal- and differential-weighted scores for LOC and OC skills in our sample indicates that differential weighting preserved the original measurement structure.

While equal-weighted LOC and OC scoring is widely used, the use of differential-weighted skills has been suggested for research studies to help mitigate potential biases and provide more insight into the skill–PA relationship [11]. Studies using equal-weighted scoring have consistently found a positive relationship between overall gross motor competence and participation in MVPA. Specifically, the association between OC skills and MVPA is non-linear, with scores below the proficiency threshold weakly related to PA [38]. In contrast, the relationships between LOC skills and MVPA are linear across all skill scores and not just the highest levels of proficiency. Regarding sex-based differences in LOC scores, a stronger relationship between LOC skills and VPA in boys than in girls was reported [14,38]. To date, reports examining relationships between equal- and differential-weighted TGMD-2 outputs for LOC and OC skills with PA outputs and HRF are lacking, likely because most studies adhere to equal-weighted scoring to maintain comparability with TGMD-2 norms and the prior literature [1,5,9,11,14]. The results of our analysis showed minimal differences for R^2^ and aR^2^ with equal- and differential-weighted skill scores when transitioning from two, five, and eight predictors for PAEE and MVPA. Similar results were found when predicting equal- and differential-weighted scores for OC; however, predicting LOC showed very little change in R^2^ and aR^2^ as predictors were added. Correlations between LOC and other variables were marginal and did not account for a similar aR^2^ as evidenced by the <1.5 Variance Inflation Factor. In summary, regression models showed that equal-weighted LOC and OC scores are adequate to make an inference on the relationships with PAEE and MVPA.

It has been discussed that the addition of more predictors generally decreases the precision of individual β coefficient estimates [39]. Specifically, our analysis showed a reduction in β coefficients for OC with MVPA from β = 0.283 (*p* ≤ 0.05) to 0.018 (*p* > 0.05), moving from two to eight predictors. No differences were noted for β coefficients for equal- and differential-weighted skill scores. The reduction in β coefficients for OC on MVPA may partially be explained by the increased effect of AP and/or sex (boys > girls) in suppressing or masking OC’s impact on PA intensity. The reduced impact of LOC on MVPA may be attributable to LP and BMI masking the impact of LOC on MVPA. Overall, our proposed masking or mediation results agree with previous reports for relationships between summed LOC and OC scores with PA and HRF [16,17] and further demonstrate that equal- and differential-weighted LOC and OC scores are comparable across several independent variables. Chi-square results showing that high OC was associated with high MVPA (>42% MVPA) were consistent with previous reports linking OC competence to MVPA [20,38] and mirrored our unadjusted regression in which weighted OC predicted MVPA (i.e., before including sex and BMI as covariates). Furthermore, the categorical association between MVPA and summed and individual average scores of OC skill showed no sex difference. The outcomes show that OC skill proficiency is associated with greater levels of guided active play MVPA across childhood for both boys and girls. This finding is inconsistent with previous research, which suggested a stronger association between OC skill and MVPA in boys than in girls [40]. Stratifying children by developmental stage might reveal sex-based differences in the association. Regarding health-related fitness, the associations between categorized high LP and AP, and high PAEE and MVPA, respectively, were comparable to the findings from multiple linear regression analysis of LP with PAEE and AP with MVPA, both adjusted and unadjusted for sex and BMI. However, the use of a cut-off threshold in Chi-square analysis revealed a strong, significant association between STR and PAEE, which was not evident in the regression analysis between PAEE and STR. In addition, the use of relevant cut-off thresholds for motor skills provides a practical approach to examine all associations among FMS, PA, and HRF across childhood, while identifying meaningful targets for positive skill- and health-related outcomes [39]. For example, categorization revealed that children aged 5–10 years may require a high STR (>24 kg) to exhibit high PAEE (>221 kcal/min^−1^). By classifying FMS, HRF, and PA into high and low groups, this method reveals performance thresholds that are associated with FMS proficiency, as well as higher levels of PA and fitness.

Additionally, we observed both sum- and average-based classifications to verify whether associations are sensitive to the scoring method. The use of individual average skill scores of LOC (LIA) and OC (OIA) in the categorical association tended to provide greater insight into the associations between FMS and PA output, as well as HRF. Specifically, a significant association was observed between high OIA and STR, an association that was not detected when using the summed OC skill score. Our results suggest that averaging individual OC skills followed by categorization allows for a meaningful association for OIA with muscle strength [13,15]. In contrast, the categorical associations between MVPA and both the summed OC score and OIA score were significant. This indicates that the method of aggregation (sum vs. average) did not differentially affect the observed relationship with PA, highlighting the robustness of OC skill and PA intensity associations regardless of scoring method.

Summed scores for the Test of Gross Motor Development (TGMD-2) play an appropriate role in education and clinical settings; however, when focused on research outcomes, the assumption that all skills are equal becomes challenging [5,11]. In short, if all items do not contribute equally, as endorsed in validation studies, then the score for each skill matters. For example, the finding that the run skill contributes half to the LOC score compared to the equal sum score constrains our understanding of the impact of motor skills on biological and behavioral variables. While equal weighting might be useful for some purposes, it could mask important differences in the quality of movement and the underlying motor control strategies. Differential weighting, on the other hand, can better capture the variations in skill execution that are more likely to be associated with physical activity levels. Whether differential weighting can provide a more refined understanding of a child’s gross movement skill development, highlighting strengths and weaknesses in specific areas, is to be determined.

Our study has some limitations to be considered when interpreting the results. Firstly, our sample size (n = 82) may have limited the stability and generalizability of our EFA findings. Statistical power analysis showed that a sample size of 86 was necessary to achieve a power level of 0.80 for EFA. However, our sample size (n = 82) was associated with a statistical power level of 0.77, which is just shy of the desirable level of >0.80. Since the smaller sample size (82 vs. 84) may have affected the generalizability and stability of the factor structure, a split-group analysis with previous data [27] showed strong model fit statistics with comparable factor structures for the data set. Future studies with larger sample sizes and factor validation using Confirmatory Factor Analysis are required.

Second, our cohort (n = 82) comprised children (49% girls) from 5 to 10 years old, with 51% between 8 and 10 years. The full cohort was analyzed and not subjected to stratified sampling, since the formation of 2–3 smaller stratified samples would have compromised statistical power for any further analysis. Thus, the heterogeneity existing in the sample may have limited the stability and generalizability of our findings when assessing the predictability of equal- and differential-weighted motor skill scores. As a result, the multiple linear regression analysis of equal- and differential-weighted motor skill scores with PA and fitness outputs was adjusted for sex, BMI, and developmental stage (early childhood and primary school children). This multiple linear regression approach provided information on important covariates within a sample size and associated statistical power of 0.99 for our sample.

Finally, the representativeness of TGMD-2 outputs is important and may limit the generalizability of our results. To assess the potential for generalizability, a comparison of descriptive and percentile data for FMS, HRF, and PAEE outputs for children in our convenience sample was completed. The average LOC and OC subtype scores for our sample of 5–10-year-old children was compared with the US normative data [5,41]. Our observations that FMS profiles and proficiency levels of our convenience sample are consistent with other published reports support the generalizability of our findings.

## 5. Conclusions

In summary, equal-weighted scores remain appropriate for clinical/educational purposes; however, to understand the nuanced relationships between PA, FMS, and HRF across childhood in a research setting, differential weighting of skills may be prudent. In our study, equal- and differential-weighted scores were related (r = 0.99) and model fits and β coefficients were similar, indicating equal weights sufficient for overall reporting. Yet differential weighting changes how individual skills shape LOC and OC scores. Although associations with PA and HRF were comparable under equal and differential weights, differential weights reveal that specific skills may drive relationships. Practically, this means equal weighting is adequate for global associations, while differential weighting promises to be more informative for designing targeted guided active play interventions. Encouraging studies that combine differential-weighted movement skills with physiology-based PA outputs for guided active play could enhance our understanding of how movement skills contribute to biological and behavioral outcomes across childhood.

## Figures and Tables

**Table 1:** Characteristics of children’s movement skills, physical activity, and health-related fitness stratified by age stages and sex (mean ± standard deviation). Abbreviations include the following: PAEE (PA energy expenditure, kcal.55 min^−1^); MVPA (percent time in moderate-vigorous PA); AP: Aerobic Power (VO_2_max, mLO_2_ kg min^−1^); LP (Leg Power in Watts); STR (average grip strength in kg); and BMI (body mass index).

	**Total** **(n = 82)**	**All Age Stages** **(n = 82)**		**Girls Age Stages** **(n = 40)**		**Boys Age Stages** **(n = 42)**	
		**I** **5–7 years** **(n = 40)**	**II** **8–10 years** **(n = 42)**	**I** **5–7 years** **(n = 20)**	**II** **8–10 years** **(n = 20)**	**I** **5–7 years** **(n = 20)**	**II** **8–10 years** **(n = 22)**
Age	7.6 ±1.5	6.3 ±0.8	8.8 ±0.8	6.1 ±1.0	8.8 ±0.9	6.5 ±0.5	8.7 ±0.8
PAEE	207.2 ±67.9	177.9 ±44.6	235.0 ^a^ ** ±74.8	168.7 ±42.1	219.6 ±64.6	187.2 ±46.2	249.0 ±82.0
MVPA	38.9 ±9.3	39.3 ±7.6	38.5 ±10.8	37.1 ±6.5	32.5 ±9.0	41.6 ±8.3	44.0 ^b^ ** ±9.4
LOC	38 ±8	39 ±7	38 ±8	37 ±8	39 ±7	40 ±6	37 ±9
OC	37 ±7	35 ±8	39 ^a^ * ±6	33 ±8	35 ±3	37 ±7	42 ^b^ ** ±5
AP	45.1 ±4.3	47.1 ±2.8	43.8 ^a^ * ±4.8	47.6 ±1.8	41.6 ±3.8	46.7 ±3.5	45.7 ^c^ * ±4.5
LP	654.4 ±386.7	514.1 ±308.1	788.0 ^a^ ** ±409.4	549.6 ±360.7	774.2 ±450.8	478.6 ±249.2	800.6 ±378.1
STR	18.8 ±10.0	9.6 ±2.6	27.5 ^a^ ** ±6.0	9.4 ±2.5	28.1 ±6.0	9.8 ±2.8	27.0 ±6.0
BMI	18.4 ±3.6	17.8 ±3.2	19.0 ±3.8	17.9 ±3.0	17.9 ±3.1	17.8 ±3.3	20.0 ±4.3

Statistical differences between stages I and II are designated with ^a^; girls vs. boys not stage-dependent are designated with ^b^; girls vs. boys across stages I and II are designated with ^c^. Probability levels are represented by * *p* ≤ 0.05; and ** *p* ≤ 0.001.

**Table 2:** Pearson Correlation Matrix for body mass index (BMI), Aerobic Power (AP), Leg Power (LP), grip strength (STR), gross motor quotient (GMQ), locomotor skills (LOC), object control skills (OC), Physical Activity Energy Expenditure (PAEE), and Moderate-to-Vigorous Physical Activity (MVPA) (n = 82).

	BMI	AP	LP	STR	GMQ	LOC	OC	PAEE
AP	−0.35 **							
LP	0.35 **	−0.23 *						
STR	0.30 **	−0.47 **	0.51 **					
GMQ	−0.11	0.21	0.17	0.08				
LOC	−0.06	0.19	0.11	−0.03	0.88 **			
OC	−0.07	0.15	0.25 *	0.22 *	0.88 **	0.570 **		
PAEE	0.55 **	−0.33 **	0.55 **	0.51 **	0.05	0.15	0.19	
MVPA	−0.10	0.45**	0.07	−0.08	0.37 **	0.28 *	0.36 **	0.67 **

*. Correlation is significant at the 0.05 level (two-tailed). **. Correlation is significant at the 0.01 level (two-tailed).

**Table 3:** Factor weightings following Exploratory Factor Analysis using Maximum Likelihood extraction and oblimin rotation for 12 locomotor and object control skills assessed by the Test of Gross Motor Development-2. The stability of the LOC and OC factor weights was compared with a split-group sample for Factor 1 and Factor 2 loadings. Standardized beta coefficients for the two-factor structure used to compute differential factor weighting are depicted in bold.

EFA Hypothesis			Split Sample		
Skill	Factor 1	Factor 2	Skill	Factor 1	Factor 2
Run	**0.555**	0.134	Run	**0.561**	0.061
Gallop	−0.088	**0.687**	Gallop	−0.172	**0.775**
Hop	**0.400**	0.208	Hop	**0.511**	0.253
Leap	0.332	**0.521**	Leap	0.402	**0.511**
Jump	0.326	**0.513**	Jump	0.228	**0.524**
Slide	0.310	**0.484**	Slide	0.050	**0.547**
Strike	**0.525**	0.061	Strike	**0.724**	−0.338
Dribble	**0.628**	0.338	Dribble	**0.475**	0.426
Catch	**0.530**	0.130	Catch	**0.515**	0.192
Kick	**0.453**	0.151	Kick	**0.492**	0.247
Throw	**0.887**	−0.160	Throw	**0.703**	−0.146
Roll	**0.734**	−0.200	Roll	**0.434**	0.102

**Table 4:** Comparison of Total, locomotor (LOC), and object control (OC) scores for equal (E)-weighted and differential (DF)-weighted Test of Gross Motor Development-2 outputs. Exploratory Factor Analysis was used to compute differential (DF)-weighted scores for Total, LOC, and OC outputs. Results and standard deviation are provided for all children (n = 82), girls (n = 40) and boys (n = 42).

	All		Girls		Boys	
Skill	E	DF	E	DF	E	DF
Total	75 ± 13	42 ± 8 *	72 ± 13	40 ± 7 *	79 ± 11 ^a^	44 ± 7 *
LOC	38 ± 8	22 ± 4 *	37 ± 7	22 ± 4 *	38 ± 8	22 ± 5 *
OC	37 ± 8	20 ± 4 *	34 ± 7	18 ± 4 *	39 ± 7 ^a^	22 ± 4 * ^a^

Statistical comparisons are noted by * for E vs. DF at *p* ≤ 0.05 and G vs. B by ^a^ at *p* ≤ 0.05.

**Table 5:** Summary of multiple linear regression analysis comparing equal and differential weighting for locomotor (LOC) and object control (OC) skills, Physical Activity Energy Expenditure (PAEE), Moderate-to-Vigorous Physical Activity (MVPA), and health-related fitness. LOC and OC skills were predicted from two (PAEE, MVPA), five (PAEE, MVPA, Aerobic Power (AP), Leg Power (LP), and average grip strength (STR)) and eight (PAEE, MVPA, AP, LP, STR, sex, body mass index (BMI), and stage) independent variables. PAEE and MVPA dependent variables were predicted from two (LOC, OC), five (LOC, OC, AP, LP, STR), and eight (LOC, OC, AP, LP, STR, sex, BMI, and stage) independent variables. Model fit measures include F-statistic (F), degrees of freedom (df), the variance accounted by R-squared (R^2^) and adjusted variance explained (aR^2^). Sex includes girls as the reference group. Statistical significance probability levels are included (*p*).

	Dependent Variables			Dependent Variables	
	Equal Weighting			Differential Weighting	
	LOC	OC		LOC	OC
Predictors: PAEE, MVPA	F = 4.73, df 2, R^2^ 0.11 aR^2^ 0.08 *p* ≤ 0.05	F = 6.78, df 2, R^2^ 0.15 aR^2^ 0.13 *p* ≤ 0.01	Predictors: PAEE, MVPA	F = 4.50, df 2, R^2^ 0.10 aR^2^ 0.08 *p* ≤ 0.05	F = 6.75, df 2, R^2^ 0.15 aR^2^ 0.12 *p* ≤ 0.01
Predictors: PAEE, MVPA, AP, LP, STR	F = 2.39, df 5, R^2^ 0.14 aR^2^ 0.08 *p* ≤ 0.05	F = 4.40, df 5, R^2^ 0.24 aR^2^ 0.17 *p* ≤ 0.001	Predictors: PAEE, MVPA, AP, LP, STR	F = 2.16, df 5, R^2^ 0.12 aR^2^ 0.07 *p* > 0.05	F = 4.21, df 5, R^2^ 0.22 aR^2^ 0.17 *p* ≤ 0.01
Predictors: PAEE, MVPA, AP, LP, STR, Sex, BMI, Stage	F = 1.74, df 7, R^2^ 0.14 aR^2^ 0.06 *p* > 0.05	F = 4.70, df 7, R^2^ 0.31 aR^2^ 0.24 *p* ≤ 0.001	Predictors: PAEE, MVPA, AP, LP, STR, Sex, BMI, Stage	F = 1.61, df 7, R^2^ 0.13 aR^2^ 0.05 *p* > 0.05	F = 4.67, df 7, R^2^ 0.31 aR^2^ 0.24 *p* ≤ 0.001
	PAEE	MVPA		PAEE	MVPA
Predictors: LOC, OC	F = 3.55, df 2, R^2^ 0.08 aR^2^ 0.06 *p* ≤ 0.05	F = 6.66, df 2, R^2^ 0.14 aR^2^ 0.12 *p* ≤ 0.01	Predictors: LOC, OC	F = 2.60, df 2, R^2^ 0.06 aR^2^ 0.04 *p* > 0.05	F = 6.89, df 2, R^2^ 0.15 aR^2^ 0.13 *p* < 0.01
Predictors: LOC, OC, AP, LP, STR	F = 10.29, df 5, R^2^ 0.40 aR^2^ 0.36 *p* ≤ 0.001	F = 6.71, df 5, R^2^ 0.26 aR^2^ 0.26 *p* ≤ 0.001	Predictors: LOC, OC, AP, LP, STR	F = 10.01, df 5, R^2^ 0.40 aR^2^ 0.36 *p* ≤ 0.001	F = 6.77, df 5, R^2^ 0.31 aR^2^ 0.26 *p* ≤ 0.001
Predictors: LOC, OC, AP, LP, STR, Sex, BMI, Stage	F = 12.00, df 7, R^2^ 0.53 aR^2^ 0.49 *p* ≤ 0.001	F = 7.08, df 7, R^2^ 0.40 aR^2^ 0.34 *p* ≤ 0.001	Predictors: LOC, OC, AP, LP, STR, Sex, BMI, Stage	F = 11.90, df 7, R^2^ 0.53 aR^2^ 0.49 *p* ≤ 0.001	F = 7.12, df 7, R^2^ 0.40 aR^2^ 0.35 *p* ≤ 0.001

**Table 6:** Chi-square and cross-tabulation analyses with effect sizes for relationships among physical activity (PA), Fundamental Movement Skills (FMS), and health-related fitness (HRF) (N = 82). MVPA = Moderate-to-Vigorous Physical Activity; PAEE = Physical Activity Energy Expenditure; OC = object control; OIA = individual average score of OC skills; STR = strength; LP = Leg Power; AP = Aerobic Power; H = high; L =low. Significance at *p* ≤ 0.05.

**Associations**	***X*** **^2^ (df = 4)**	**Cramer’s V**	**Overexpression Observed in H and L Categories**
PA and FMS			
MVPA with OC	9.42	0.24	37% of H OC in H MVPA
MVPA with OIA	9.55	0.24	37% of H OIA in H MVPA
PA and HRF			
PAEE with LP	14.04	0.29	52% of H LP in H PAEE; 57% of L LP in L PAEE
PAEE with STR	15.51	0.30	56% of H STR in H PAEE
MVPA with AP	14.55	0.31	57% of L AP in L MVPA
HRF and FMS			
STR with OIA	11.21	0.25	42% of H STR in H OIA

## Data Availability

The datasets generated during and/or analyzed during the current study are not publicly available but are available from the corresponding author, who was an organizer of the study.

## Supplementary Materials

The following supporting information can be downloaded at: https://www.mdpi.com/article/10.3390/children12121615/s1. Table S1: Standardized β coefficients for Physical Activity Energy Expenditure (PAEE) and Moderate-Vigorous Physical Activity (MVPA) predicted by equal-weighted locomotor (LOC) and object control (OC) skills, Aerobic Power (AP), Leg Power (LP), strength (STR), sex, and BMI: two-, five-, and eight-factor predictor models; Table S2: Standardized β coefficients for Physical Activity Energy Expenditure (PAEE) and Moderate-Vigorous Physical Activity (MVPA) predicted by differential-weighted (DF) locomotor (LOC) and object control (OC) skills, Aerobic Power (AP), Leg Power (LP), strength (STR), sex, and BMI: two-, five-, and seven-factor predictor models; Table S3: Standardized β coefficients for equal-weighted locomotor (LOC) and object control (OC) skills predicted by Physical Activity Energy Expenditure (PAEE) and Moderate-Vigorous Physical Activity (MVPA), Aerobic Power (AP), Leg Power (LP), strength (STR), sex, and BMI: two-, five-, and seven-factor predictor models; Table S4: Standardized β coefficients for differential-weighted locomotor (LOC) and object control (OC) skills predicted by Physical Activity Energy Expenditure (PAEE) and Moderate-Vigorous Physical Activity (MVPA), Aerobic Power (AP), Leg Power (LP), strength (STR), sex, and BMI: two-, five-, and seven-factor predictor models.
