# Supplementary material for: Computing and Evaluating Relationships Between Equal and Differential Factor Weighting for Fundamental Movement Skills and Physical Activity with Guided Active Play During Childhood
Source: Children (Basel). 2025 Nov 27;12(12):1615. doi: 10.3390/children12121615 (PMC12731510; doi:10.3390/children12121615)
Supplement: Supplementary file 1 [file children-12-01615-s001.zip › children-3915369-supplementary.pdf]

Table S1: Standardized β Coefficients for Physical Activity Energy Expenditure (PAEE) and Moderate-Vigorous Physical Activity (MVPA) Predicted by Equal-Weighted Locomotor (LOC) and Object Control (OC) Skills, Aerobic Power (AP), Leg Power (LP), Strength (STR), Sex, BMI and Stage: Two, Five, and Eight Factor Predictor Models.

Table S1-E

Independent Variables

Dependent (Outcome) Variables	**LOC β CI95% (L, H)**	**OC β CI95% (L, H)**	**AP β CI95% (L, H)**	**LP β CI95% (L, H)**	**STR β CI95% (L, H)**	**Sex β CI95% (L, H)**	**BMI β CI9% (L, H)**	**Stage β CI% (L, H)**
PAEE	−0.263 (−4.775, 0.019) p>0.05	0.339 (0.708, 5.454) p≤0.05						
MVPA	0.139 (-0.142, 0.490) p>0.05	0.283 (0.038, 0.668) p≤0.05						
PAEE	−0.149 (−3.376, 0.657) p>0.05	0.153 (−0.726 3.505) p>0.05	−0.136 (−5.495, 1.173) p>0.05	0.385 (0.031, 0.104) p≤0.05	0.210 (−0.180, 3.008) p>0.05			
MVPA	0.070 (-0.211, 0.387) p>0.05	0.225 (-0.033, 0.594) p>0.05	0.441 (0.470, 1.458) (p≤0.05	0.091 (-0.003, 0.008) p>0.05	0.035 (-0.204, 0.268) p>0.05			

PAEE	-0.080 (-2.950, 1.359) $p>0.05$	0.116 (-1.178 3.126) $p>0.05$	-0.095 (-4.501, 1.683) $p>0.05$	0.293 (0.013, 0.084) $p\leq 0.05$	0.038 (-2.516, 2.989) $p>0.05$	0.149 (-6.466, 43.643) $p>0.05$	0.316 (2.282, 9.615 $p\leq 0.001$	0.184 (-26.724, 72.588) $p>0.05$
MVPA	0.174 (-0.086, 0.601) $p>0.05$	0.012 (-0.328, 0.358) $p>0.05$	0.376 (0.337, 1.324) $p\leq 0.05$	0.122 (-0.003, 0.009) $p>0.05$	-0.048 (-0.484, 0.396) $p>0.05$	0.351 (2.558, 10.550) $p\leq 0.05$	-0.049 (-0.724, 0.446) $p>0.05$	0.106 (-5.943, 9.896) $p>0.05$

Table S2: Standardized β Coefficients for Physical Activity Energy Expenditure (PAEE) and Moderate-Vigorous Physical Activity (MVPA) Predicted by Differential-Weighted (DF) Locomotor (LOC) and Object Control (OC) Skills, Aerobic Power (AP), Leg Power (LP), Strength (STR), Sex, BMI and Stage: Two, Five, and Eight Predictor Models.

Table S2-DF	*Independent Variables*							
Dependent (Outcome) Variables	**LOC β CI95% (L, H)**	**OC β CI95% (L, H)**	**AP β CI95% (L, H)**	**LP β CI95% (L, H)**	**STR β CI95% (L, H)**	**Sex β CI95% (L, H)**	**BMI β CI95% (L, H)**	**Stage β CI95% (L, H)**
PAEE	−0.219 (−7.416, 0.638) $p>0.05$	0.289 (0.464, 8.776) $p\leq 0.05$						
MVPA	0.136 (-0.238, 0.817)	0.294 (0.101, 1.190) $p\leq 0.05$						

	$p>0.05$							
PAEE	−0.113 (−5.123, 1.628) $p>0.05$	0.121 (−1.721 5.587) $p>0.05$	0.222 (−5.507, 1.196) $p>0.05$	0.386 (0.031, 0.104) $p\leq0.05$	0.251 (−0.097, 3.008) $p>0.05$			
MVPA	0.076 (-0.336, 0.658) $p>0.05$	0.225 (-0.044, 1.003) $p>0.05$	0.440 (0.467, 1.454) $p\leq0.05$	0.094 (-0.003, 0.008) $p>0.05$	0.038 (-0.199, 0.270) $p>0.05$			
PAEE	-0.081 (-4.794, 2.098) $p>0.05$	0.127 (-1.835, 5.641) $p>0.05$	-0.096 (-4.504, 1.659) $p>0.05$	0.284 (0.012, 0.083) $p\leq0.01$	0.041 (-2.486, 3.005) $p>0.05$	0.142 (-7.454, 42.947) $p>0.05$	0.320 (2.351, 9.685 $p\leq0.001$	0.179 (-27.285, 71.995) $p>0.05$
MVPA	0.166 (-0.137, 0.964) $p>0.05$	0.024 (-0.544, 0.651) $p>0.05$	0.378 (0.343, 1.328) $p\leq0.05$	0.126 (-0.003, 0.009) $p>0.05$	-0.044 (-0.480, 0.398) $p>0.05$	0.348 (2.467, 10.521) $p\leq0.05$	-0.054 (-0.739, 0.433) $p>0.05$	0.100 (-6.072, 9.793) $p>0.05$

Table S3: Standardized β Coefficients for Equal-Weighted Locomotor (LOC) and Object Control (OC) Skills Predicted by Physical Activity Energy Expenditure (PAEE) and Moderate-Vigorous Physical Activity (MVPA), Aerobic Power (AP), Leg Power (LP), Strength (STR), Sex, BMI, and Stage: Two, Five, and Eight Factor Predictor Models.

Table S3-E	*Independent Variables*							
Dependent (Outcome) Variables	**PAEE β CI95% (L, H)**	**MVPA β CI95% (L, H)**	**AP β CI95% (L, H)**	**LP β CI95% (L, H)**	**STR β CI95% (L, H)**	**Sex β CI95% (L, H)**	**BMI β CI95% (L, H)**	**Stage β CI95% (L, H)**
LOC	-0.130 (-0.038, 0.009) p>0.05	0.325 (0.088, 0.432) p≤0.05						
OC	0.125 (-0.009, 0.034) p>0.05	0.339 (0.130, 0.440) p≤0.05						
LOC	-0.219 (-0.056, 0.008) p>0.05	0.304 (0.035, 0.451) p≤0.05	0.054 (-0.404, 0.552) p>0.05	0.200 (-0.001, 0.009) p>0.05	0.002 (-0.207, 0.209) p>0.05			
OC	-0.030 (-0.033, 0.027) p>0.05	0.306 (0.048, 0.443) p≤0.05	0.161 (-0.192, 0.756) p>0.05	0.144 (-0.002, 0.008) p>0.05	0.265 (0.001, 0.394) p≤0.05			
LOC	-0.146 (-0.047, 0.017)	0.307 (0.009, 0.406)	0.032 (-0.395, 0.490)	0.182 (-0.002, 0.008)	0.087 (-0.303, 0.413)	0.017 (-2.985, 3.432)	0.043 (-0.428, 0.592)	-0.046 (-7.036, 5.851)

	$p>0.05$	$p\leq0.05$	$p>0.05$	$p>0.05$	$p>0.05$	$p>0.05$	$p>0.05$	$p>0.05$
OC	0.034 (-0.029, 0.037) $p>0.05$	0.128 (-0.101, 0.305) $p>0.05$	0.109 (-0.260, 0.645) $p>0.05$	0.217 (-0.001, 0.009) $p>0.05$	0.099 (-0.292, 0.440) $p>0.05$	0.345 (1.867, 8.415) $p\leq0.05$	-0.128 (-0.810, 0.233) $p>0.05$	0.156 (-4.271, 8.930) $p>0.05$

Table S4: Standardized β Coefficients for Differential-Weighted Locomotor (LOC) and Object Control (OC) Skills Predicted by Physical Activity Energy Expenditure (PAEE) and Moderate-Vigorous Physical Activity (MVPA), Aerobic Power (AP), Leg Power (LP), Strength (STR), Sex, BMI, and Stage: Two, Five, and Eight Factor Predictor Models.

Table S4-DF	*Independent Variables*							
Dependent (Outcome) Variables	**PAEE β CI95% (L, H)**	**MVPA β CI95% (L, H)**	**AP β CI95% (L, H)**	**LP β CI95% (L, H)**	**STR β CI95% (L, H)**	**Sex β CI95% (L, H)**	**BMI β CI95% (L, H)**	**Stage β CI95% (L, H)**
LOC	-0.120 (-0.022, 0.006) p>0.05	0.320 (0.049, 0.263) p≤0.01						
OC	0.103 (-0.007, 0.020) p>0.05	0.349 (0.063, 0.254) p≤0.001						
LOC	-0.192 (-0.031, 0.006) p>0.05	0.298 (0.017, 0.263) p≤0.05	0.052 (-0.242, 0.348) p>0.05	0.174 (-0.001, 0.005) p>0.05	−0.003 (-0.207, 0.121) p>0.05			
OC	-0.043 (-0.020, 0.015) p>0.05	0.315 (0.031, 0.256) p>0.05	0.159 (-0.113, 0.428) p>0.05	0.137 (-0.001, 0.00)4 p>0.05	0.252 (-0.006, 0.219) p>0.05			

LOC	-0.151<br>(-0.028,<br>0.010)<br>p>0.05	0.308<br>(0.005,<br>0.243)<br>p≤0.05	0.016<br>(-0.251,<br>0.280)<br>p>0.05	0.147<br>(-0.001,<br>0.004)<br>p>0.05	0.065<br>(-0.190,<br>0.239)<br>p>0.05	0.011<br>(-1.835,<br>2.006)<br>p>0.05	0.081<br>(-0.214,<br>0.397)<br>p>0.05	-0.027<br>(-4.073,<br>3.671)<br>p>0.05
OC	0.053<br>(-0.059,<br>0.022)<br>p>0.05	0.118<br>(-0.059,<br>0.164)<br>p>0.05	0.100<br>(-0.151,<br>0.348)<br>p>0.05	0.239<br>(0.000,<br>0.005)<br>p≤0.05	0.048<br>(-0.182,<br>0.222)<br>p>0.05	0.361<br>(1.201,<br>4.815)<br>p≤0.05	-0.130<br>(-0.451,<br>0.125)<br>p>0.05	0.186<br>(-2.091,<br>5.195)<br>p>0.05
